# Yi-qi-yang-yin-tian-sui-fang enhances cisplatin-induced tumor eradication and inhibits interleukin-7 reduction in non-small cell lung cancer

**DOI:** 10.1042/BSR20190052

**Published:** 2019-06-28

**Authors:** Bin Ke, Xinlin Wu, Qiong Yang, Yuanyuan Huang, Fang Wang, Yuxin Gong, Junfang Liu, Lin Shi

**Affiliations:** 1 Department of Traditional Chinese Medicine, The First Affiliated Hospital of Sun Yat-sen University, Guangzhou, Guangdong; 2 Department of Oncology, Sun Yat-sen Memorial Hospital of Sun Yat-sen University, Guangzhou, Guangdong; 3 Department of VIP Ward, Affiliated Cancer Hospital of Sun Yat-Sen University, Guangzhou, Guangdong; 4 Department of Oncology, The First Affiliated Hospital of Sun Yat-sen University, Guangzhou, Guangdong; 5 Department of Respiratory, Zhujiang Hospital of Southern Medical University, Guangzhou, Guangdong; 6 Department of Traditional Chinese Medicine, Zhujiang Hospital of Southern Medical University, 253 Industrial Road, Guangzhou, Guangdong 510280, People’s Republic of China

**Keywords:** Chemotherapy, Cisplatin (DDP), IL-7, Myelosuppression, NSCLC, Traditional Chinese Medicine (TCM)

## Abstract

Traditional Chinese Medicine (TCM) has been recognized to be conducive to enhancing the efficiency and reducing the side effects in the whole course of cancer treatment. The mechanisms of TCM/chemotherapy combination involved with interleukin-7 (IL-7) potentially enhance immune responses against tumor. In the present study, we emphasized on a herbal formulation Yi-qi-yang-yin-tian-sui-fang or TCM for short, and investigated its roles in chemotherapy in non-small cell lung cancer (NSCLC). The mice bared with tumor were treated with cisplatin (DDP) and simultaneously administrated with/without low, medium and high doses of TCMs (effective content: 0.5, 2.0 and 8.0 g/per mice) via oral gavage. The results indicated that combination of TCM further elevated the therapy efficiency of DDP in a dose-dependent manner. The growth of tumor cells was estimated by Ki-67 stain and terminal deoxynucleotidyl transferase-mediated dUTP-biotin nick-end labeling (TUNEL) assay. The addition of TCM to the DDP treatment could significantly decrease the expression of Ki-67 and promote the apoptosis of tumor cells. In addition, the serum IL-7 level was down-regulated by DDP but restored by the treatment of TCM. The expression of IL-7 and its receptor IL-7R in tumor tissues was also recovered by TCM. Furthermore, the side effect from bone marrow suppression (myelosuppression) induced by DDP were assessed. TCM could abrogate DDP-induced apoptosis of bone marrow and also remarkably induced the expressions of IL-7 and hematopoietic growth factors including G-CSF, GM-CSF, SCF, and SDF-1 in bone marrow. These data indicated that this TCM combined with DDP showed superior anti-tumor effects with reduced myelosuppression via up-regulating IL-7.

## Introduction

As a global health burden, lung cancer accounts for 19.4% of cancer-related deaths with 1.59 million deaths annually, and remains the leading cause of cancer-related deaths worldwide. Lung cancers are pathologically classified into non-small cell lung carcinoma (NSCLC) (accounting for 80%) and small cell lung carcinoma (SCLC) (accounting for 20%) [1,2].Although the platinum-based doublet chemotherapy with or without targeted therapies have been evaluated in the treatment of NSCLC, the improvements of clinical outcomes have largely been confined to patients with adenocarcinoma due to the efficiency issues and/or side effects including cancer-related fatigue, pain, nausea, vomiting, nephrotoxicity, lymphopenia and myelosuppression [3–5]. Therefore, new therapies for the management of advanced lung cancer are needed and should be suitable for improving quality of life and permitting long-term use.

The addition of Traditional Chinese Medicines (TCM) as maintenance therapy into conventional radiotherapy and chemotherapy is recommended for all kinds of patients, including patients with cancer [6]. The treatment of TCM could not only enhance the efficacy of conventional therapy but also reduce the side effects and complications caused by chemo- and radiotherapy [7]. For example, in colorectal cancer, the Huang-qi compounds could activate the immune system with increased proportions of T-lymphocyte subsets and reduce the incidence of nausea, vomiting and leukopenia in patients treated with chemotherapy [8]. Shi-quan-da-bu-tang (TJ-48) is a well-known Chinese herbal formulation. The TJ-48 was able to attenuate liver Kupffer cell-induced oxidative stress in patients with hepatocellular carcinoma and improve hepatic recurrence-free survival [9]. Besides, it has been demonstrated that Shen-qi-fu-zheng injection can prolong the survival time, improve the quality of life, and reduce the side effects for advanced NSCLC [10]. Our ongoing clinical study investigated a herbal formulation Yi-qi-yang-yin-tian-sui-fang, and found that this TCM could reduce nephrotoxicity, bone marrow suppression after chemotherapy and improve the body’s immune function (unpublished data).

The immune system is critical for cancer surveillance and therapy. The bone marrow, spleen and thymus are main immune organs but radio-chemotherapy often destroys the cells of these organs and decreases lymphocyte count, leading to treatment-related lymphopenia and myelosuppression [11,12]. Interleukin-7 (IL-7) is a non-hematopoietic cell-derived cytokine required for T-cell development and for maintaining and restoring homeostasis of mature T cells. IL-7 is mainly produced by non-hematopoietic cells including keratinocytes in the skin, fibroblastic stromal cells in the bone marrow and is up-regulated to increase T-cell proliferation during lymphopenia in many diseases, such as HIV, hepatitis B virus and hepatitis C virus infections [13,14]. Therefore, recombinant human IL–7 (rhIL-7) has been produced for treating patients with cancer. Gou et al. [15] reported that the administration of IL-7 combined with oxaliplatin effectively inhibited the growth of tumors in lung and abdomen metastasis models of colon cancer, which was associated with up-regulation of tumor-infiltrating activated CD8^+^ T cells and down-regulation of regulatory T (Treg) cells in spleen. Interestingly, Ayeka et al. [16] reported that the expression of IL-7 could be elevated by some TCMs for activating immune cells and suppressing tumor growth. Currently, the function of IL-7 in NSCLC remains to be clarified.

In the present study, we focused on the herbal formulation Yi-qi-yang-yin-tian-sui-fang, TCM for short, and investigated the roles of this TCM and IL-7 in chemotherapy (cisplatin, DDP) of NSCLC. We speculated that this TCM could up-regulate the IL-7 level and enhance the efficiency of DDP while reduce the bone marrow suppression.

## Materials and methods

### Cell lines and reagents

The NSCLC cell line A549 was purchased from the Cell Bank of the Chinese Academy of Sciences (Shanghai, China) and cultured in Dulbecco’s modified Eagle’s medium (DMEM) supplemented with 10% FBS (Life Technologies, U.S.A.), ampicillin and streptomycin at 37°C, 5% CO_2_ conditions. The Yi-qi-yang-yin-tian-sui-fang (TCM) consisted of antler gum 15 g, turtle shell (first fried 40 min) 50 g, American ginseng (*Panax quinquefolius* Linn.) 15 g, medlar (*Lycium chinense* Miller) 15 g, donkey-hide gelatin (*Colla corii asini*) 15 g, ginseng (*Panax ginseng* C.A. Meyer) 30 g, astragalus 30 g, angelica (*Angelica sinensis* (Oliv.) Diels) 6 g, pseudo-ginseng (*Stahlianthus involucratus* (King ex Bak.) Craib ex Loesener) 10 g, which was prepared by conventional decoction, water bath concentration, and was stored at 4.0°C.

### Preparation of drug-containing serum

The drug used in this study is a drug-containing serum and is used to treat NSCLC. In the present study, a total of 120 Wistar rats weighing 120–150 g were randomly divided into four groups and fed standard diet. The low dose, medium dose and high dose groups were intragastrically administered saline containing equivalently 10.5, 42, and 168 g/l TCM, respectively, while the control group was fed with saline of the same volume and were fed once a day for 7 days. Blood from different groups (10 ml) was aseptically obtained from the abdominal aorta of the rats 2 h after the last feeding and immediately centrifuged at 2000×***g*** for 10 min at 4°C to harvest the serum. The preparation of drug-containing serum was blended according to different groups and stored at −70°C, inactivated at 56°C before use. The approximate concentration of TCM in blood was 1.05, 4.2 and 16.8 mg/10 ml, namely 0.1, 0.4 and 1.6 mg/ml.

### Establishing tumor model and the TCM/DDP treatment

In the present study, a total of 36 male nude mice weighing 25–30 g were provided by Animal Center of Southern Medical University according to its Ethics Committee and the ethics approval had been obtained from. The xenograft model of human A549 was established and 2 × 10^6^ A549 cells were subcutaneously injected in rear flank of 5–6 weeks old nude mice. All the treatment of DDP in related groups began 5 days after subcutaneous tumor cells injection and the nude mice were treated i.p with DDP daily at 5 mg/kg for 7 days. In the treatment of TCM alone group, 0.5 ml high dose of TCM (8.0 g/per mice) was administrated daily for 22 days after subcutaneous tumor cells injection. In the combination groups, apart from the treatment of DDP, three doses of 0.5 ml TCM, low, medium and high dose (effective content: 0.5, 2.0 and 8.0 g/per mice) were administrated daily for 22 days. Tumor volume was measured weekly after 7 days from tumor cells injection. At day 28, the mice were killed by twisting the cervical spine. The tumors were collected for the measurement of weight. The tumor volumes were estimated using the following formula: tumor volume (cm^3^) = (length*width ^2^)/2.

### Hematoxylin and Eosin staining

The tumor tissue samples were fixed with 4% paraformaldehyde immediately for 24 h. Then fixed samples were washed and gradient dehydrated by different levels of ethanol. After hyalinization with xylene, the samples were embedded in paraffin overnight and sliced into 4-µm thick consecutive sections. Sections were baked, dewaxed and hydrated. Eventually, they were stained with Hematoxylin and Eosin (HE) for histological observation.

### Immunohistochemistry

For the immunohistochemical assay, tumor sections were incubated at 60°C for 2 h before incubation with dimethylbenzene for dewaxing, and washed with PBS for three times, followed by incubation with a primary antibody against Ki-67 at 37°C for 30 min and then at 4°C overnight. After three washes with 0.01 M PBS (5 min each), a secondary antibody was added to the sections for 30-min incubation at 37°C, followed by five cycles of PBS wash. Thereafter, the sections were incubated with HRP-labeled avidin for 30 min at 37°C, and reacted with DAB.

### Terminal deoxynucleotidyl transferase-mediated dUTP-biotin nick-end labeling assay

Sections of tumor tissues and bone marrow were stained by terminal deoxynucleotidyl transferase-mediated dUTP-biotin nick-end labeling (TUNEL) method, using apoptosis *in situ* detection kit (Wako Pure Chemical, Osaka, Japan), according to the instructions. The ratio of TUNEL-positive cell number to the total cell number is shown.

### Enzyme-linked immunosorbent assay

The concentrations of IL-7 in serum and bone marrow were determined by enzyme-linked immunosorbent assay (ELISA) kit (R&D Systems, U.S.A.) according to the manufacturer’s manual. The detection range of this kit is 31.2–2000 pg/ml and the sensitivity is 8.3 pg/ml.

### Quantitative real-time PCR analysis

Total RNA was extracted from bone marrow using TRIzol reagent (Invitrogen, U.S.A.). cDNA synthesis was performed using a PrimeScript RT Reagent Kit (Takara, China) according to the manufacturer’s manual. The PCR amplification was performed with the conditions of 95°C for 10 s, 40 cycles of 94°C for 30 s, 60°C for 30 s, and 72°C for 30 s on an ABI 7900 system (Applied Biosystems, U.S.A.) with SYBR Green Real-time PCR Master Mix (Takara, China). Expression levels of genes were internally normalized to that of the GAPDH. The relative levels of genes were calculated by the 2^−ΔΔ*C*^_t_ method. Each experiment was performed in triplicate.

### Western blot assay

The tumor tissues were used to extract the total proteins by lysing cells in RIPA buffer with Protease Inhibitor Cocktail (Pierce, U.S.A.). BCA Protein Assay Kit (Beyotime, China) was used to measure protein concentration. According to the manufacturer’s protocols, equivalent amounts of protein were separated by 10% SDS/PAGE polyacrylamide gels and then transferred to PVDF membranes (Millipore, U.S.A.). The membranes were blocked with 5% BSA in Tris-buffered saline and then incubated with primary antibodies followed by horseradish peroxidase–conjugated secondary antibody (Abcam, U.S.A.). The protein signals were detected by ECL chemiluminescence kit and quantitated using ImageJ software. GAPDH was used as a loading control.

### Statistical analyses

The results are analyzed by the Statistical Package for Social Sciences version 16.0 (SPSS 16.0, SPSS Inc., Chicago, IL, U.S.A.) and the Prism Statistical Software package (Version 5.0, GraphPad Software Inc.). Unpaired *t* tests or Mann–Whitney U tests were used to compare the two groups. *P*<0.05 was considered statistically significant. All experiments were performed at least three times.

## Results

### TCM could consolidate the DDP-induced tumor regression in NSCLC

**Figure 1:**
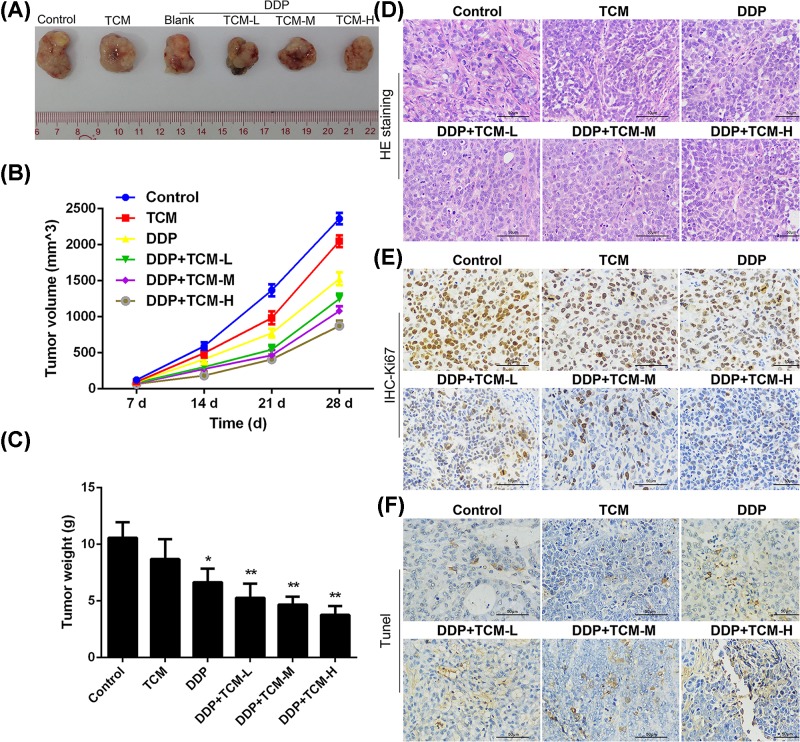
The enhanced anti-tumor role of TCM in the DDP-treated lung cancer*in vivo* A549 cells were subcutaneously injected in rear flank of nude mice (six per group) and three doses of TCM (effective content: 0.5, 2.0 and 8.0 g/per mice) were administrated with/without DDP to mice. (**A**–**C**) The mean tumor size (mm3) and tumor weight were analyzed. (**D,E**) The HE staining and Ki-67 stain by IHC were performed to determine the proliferation of tumor cells. (**F**) The apoptosis was estimated by the Tunel assay. *P<0.05, **P<0.01. Data represent the means ± s.d.

To determine the function of the TCM (Yi-qi-yang-yin-tian-sui-fang) during the chemotherapy of NSCLC, the xenograft model of human A549 was established. The results showed that DDP significantly inhibited the tumor growth but the high dose of TCM alone had no anti-tumor effect. However, the combination of three different doses of TCM and DDP showed further enhanced efficiency of tumor eradication. The mice treated with DDP combined with high dose TCM harbored the smallest tumor volume and tumor weight (Figure 1A–C). The anti-tumor effects were confirmed by HE staining and proliferation index Ki-67 stain. Increased tumor cell nucleus was inhibited by the treatment of TCM/DDP, the atypia and split image were also decreased in the tumor tissues compared with the tissues without the treatment of TCM (Figure 1D). The expression of Ki-67 was dose-dependently reduced by the combination of DDP with TCM, leading to impaired tumor proliferation. And the group of high dose TCM with DDP showed the lowest Ki-67 levels in tumor tissues (Figure 1E). Tunel apoptosis assay further confirmed that the addition of high dose TCM to DDP treatment could efficiently induce tumor cell apoptosis when compared with the DDP treatment alone (Figure 1F). The results indicated that the combination of DDP and three doses of TCM showed to inhibit the tumor proliferation in tumor tissues and promote the apoptosis of tumor cells. These data demonstrated that the TCM could enhance the efficiency of DDP for the treatment of NSCLC *in vivo*.

### The expression of IL-7/IL7R is recovered by TCM in tumor tissues and serum

**Figure 2:**
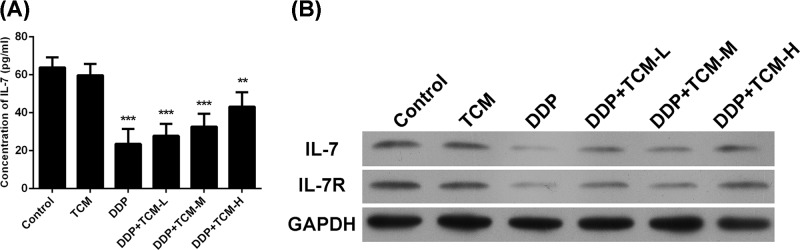
The IL-7/IL-7R are restored by TCM in tumor tissue and serum (**A**) The serum level of IL-7 was analyzed by ELISA. (**B**) The expression of IL-7/IL7R was evaluated by Western blot in tumor tissues. **P<0.01, ***P<0.001. Data represent the means ± s.d.

Since the protective role of IL-7 signal during the treatment-related lymphopenia, the serum level of IL-7 was determined by ELISA. The results showed that the treatment of DDP significantly reduced the IL-7 level in serum. However, the addition of TCM to the DDP therapy could restore the serum level of IL-7, although the TCM alone showed no significant impacts on the serum level of IL-7 (Figure 2A). Moreover, the expressions of IL-7 and its receptor IL-7R in tumor tissues were also determined by Western blot assay (Figure 2B). The results of IL-7 and IL-7R levels in tumor tissues showed the similar trends as that in serum. The combination of DDP and TCM could restore the IL-7/IL-7R signals in TCM dose-dependent manner, suggesting that DDP impaired the protective role of IL-7 during the chemotherapy but the TCM could inhibit this effect and recover the IL-7/IL7R expressions in tumor tissues.

### TCM extenuates the DDP-induced bone marrow suppression

**Figure 3:**
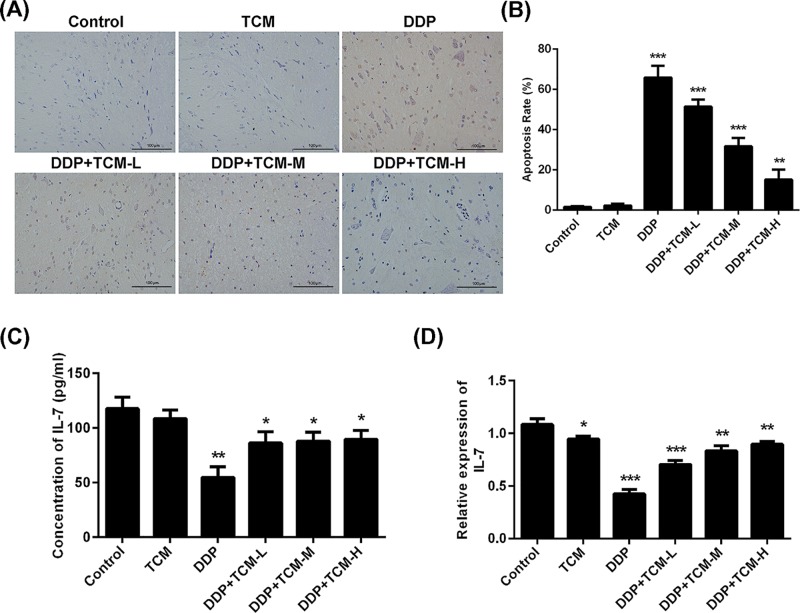
The protective effect of TCM on bone marrow (**A,B**) The TCM/DDP-related cytotoxicity of bone marrow was determined by Tunel assay. (**C,D**) The expression of IL-7 in bone marrow was determined by Q-PCR and ELISA. *P<0.05, **P<0.01, ***P<0.001. Data represent the means ± s.d.

The long-term treatment of chemotherapy in clinic would lead to many side effects and complications including the cancer-related nephrotoxicity, lymphopenia and myelosuppression. We here investigated the protective role of TCM in DDP-induced myelosuppression. The apoptosis of bone marrow tissues was estimated by Tunel apoptosis assay (Figure 3A,B). The results indicated that the treatment of DDP remarkably produced bone marrow cytotoxicity and the TCM alone has no bone marrow cytotoxicity. However, TCM abrogated the DDP-induced apoptosis of bone marrow in dose-dependent manner and the high dose of TCM could reduce this myelosuppression to the least extent.

In addition, the concentration and mRNA expression level of IL-7 were also estimated by ELISA and quantitative real-time PCR (qRT-PCR), and showed similar results. The treatment of DDP significantly inhibited the protein and mRNA expression of IL-7 in bone marrow. The addition of TCM significantly restored the DDP-induced reduction in IL-7 level in bone marrow, although the TCM alone could not increase the protective effect (Figure 3C,D).

### Bone marrow, IL-7 and hematopoietic growth factors are induced by TCM

**Figure 4:**
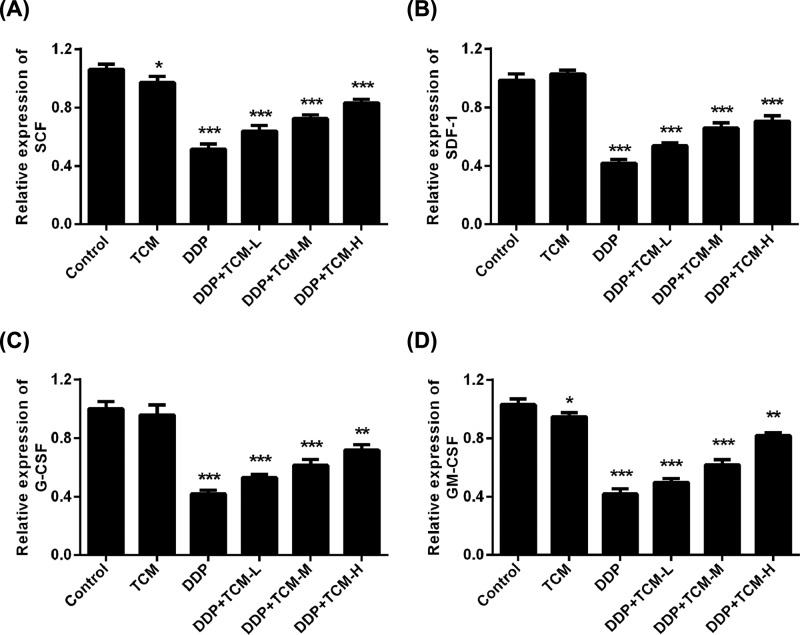
The expressions of bone marrow IL-7 and hematopoietic growth factors are induced by TCM (**A**–**D**) The hematopoietic growth factors including G-CSF, GM-CSF, SCF and SDF-1 in bone marrow were analyzed by Q-PCR. *P<0.05, **P<0.01, ***P<0.001. Data represent the means ± s.d.

Currently, there are various different strategies to prevent chemotherapy-induced myelosuppression, especially the growth factors-based primary prophylaxis prior to chemotherapy including rHuG-CSF, rHuGMCSF etc [17]. Therefore, the levels of these growth factors in bone marrow are critical for reflecting the protective of TCM during chemotherapy. The hematopoietic growth factors including SCF, SDF-1, G-CSF, GM-CSF in bone marrow were analyzed and found that the DDP-based chemotherapy significantly reduced the expression of these genes in bone marrow. However, the combined treatment of TCM effectively attenuated this inhibition and up-regulated the levels of SCF, SDF-1, G-CSF, GM-CSF in bone marrow (Figure 4A–D). These data implicated that the TCM had effective protects of bone marrow and diminished the chemotherapy-induced side effects.

## Discussion

NSCLC accounts for more than 85% of all lung cancers. Since the large proportion of NSCLC patients present with locally advanced or metastatic disease when diagnosed, the prognosis for advanced-stage disease is very poor with an overall 5-year survival rate of 17% [18]. Different improved strategies have been tested in clinical and platinum-doublet chemotherapy with or without targeted therapies contributing to the stepwise increases in survival for advanced NSCLC but with significant side effects and complications [19]. The TCM has been proposed to be applied into cancer treatment to increase the efficiency of therapy and decrease the incidence of side effects. In the present study, we investigated a TCM, Yi-qi-yang-yin-tian-sui-fang, and discovered its anti-tumor effects and bone marrow protective function in the chemotherapy of NSCLC.

TCM is an important component of complementary and alternative medicine and widely used to treat various diseases [20]. The supplements of many TCMs, such as Ginseng, Huang-qi, Ban-zhi-lian etc., to the chemo- or radiotherapy are capable of further elevating the response rate and diminishing the side effects and complications caused by chemo- and radiotherapy [6]. The combination of Huang-qi compounds and chemotherapy could improve the short-term prognosis and clinical outcome in acute lymphoblastic leukemia in children [21]. The intervention of essential oil of vetiver was found to reduce DDP-induced oxidative stress, nephrotoxicity and significantly inhibit DNA damage, clastogenic effects and cell cycle arrest in the bone marrow cells of Swiss albino mice [22]. In the present study, we utilized the TCM (Yi-qi-yang-yin-tian-sui-fang) in the chemotherapy of NSCLC and found that the addition of TCM could enhance DDP-induced inhibition of tumor growth and reduce the DDP-induced apoptosis of bone marrow cells, which was consistent with previous study. Accumulating evidences have demonstrated that the ingredient of Yi-qi-yang-yin-tian-sui-fang showed the anti-tumor effects with high safety. Donkey-hide gelatin was clearly confirmed to promote the recovery of bone marrow hemopoietic function in a myelosuppressed mouse model [23]. American ginseng was reported to exert the chemo-preventive effects by anti-inflammatory and antioxidant mechanisms [24] and Panax ginseng extract exerted a protective effect in a rat model of cachexia and suggest that Panax ginseng extract may be a therapeutic promising tool for supportive care in oncology [25]. Besides, the organic extract of Angelica can inhibit body weight decrease caused by DDP and reduce DDP toxicity due to decreasing release of LDH, AST, ALT, and AKP into blood and enhancing thymus protection [26]. But the detailed mechanism underlying these protective roles of TCM is far more unclear.

As a critical cytokine for T-cell development and homeostasis of mature T cells, IL-7 accumulates during lymphopenia, leading to increased T-cell proliferation [13]. Although the function of IL-7 in cancer has not reached the consistence, mounting evidence *in vivo* showed that the administration of rhIL-7 is beneficial for the tumor treatment via promoting the development of activation T cells, B cells and NK cells [5]. Interestingly, many TCMs were demonstrated to up-regulate the IL-7 level for the inhibition of tumor growth. The low molecular weight of *Glycyrrhiza uralensis* polysaccharides could promote the secretion of anticancer cytokine IL-7 to increase the proliferation and maturation of T lymphocytes *in vitro* and promote the activation of CD4^+^ and CD8^+^ immune cells population *in vivo* for suppressing tumor growth of CT-26 [16,27]. The flavonoids of *Hippophaerhamnoides L* (TFH) isolated from berries of sea buckthorn up-regulated the levels of IL-1α, IL-2, IL-7, IL-15 etc. to activate NK cells and its cytotoxicity against tumor cells K562 [28]. We here found that the TCM elevated the expression of IL-7 in serum and tumor or bone marrow tissues in response to the DDP-based chemotherapy of NSCLC, which is favorable to the treatment and the relief from side effects in bone marrow via up-regulation of hematopoietic growth factors including SCF, SDF-1, G-CSF, GM-CSF. These evidences demonstrated that up-regulation of IL-7 is important factor for TCM-induced therapy efficiency.

## Conclusion

In conclusion, we found that combined TCM to DDP-based chemotherapy significantly enhanced inhibition efficiency of tumor growth while reduced the chemotherapy-induced myelosuppression and IL-7 reduction, which, in turn, restored the protective function of IL-7 and hematopoietic growth factors.

## Abbreviations

DDP: cisplatin
ELISA: enzyme-linked immunosorbent assay
HE: Hematoxylin and Eosin
IL-7: interleukin-7
i.p: Intraperitoneal injection
NSCLC: non-small cell lung cancer
rhIL-7: recombinant human IL–7
RIPA: Radio Immunoprecipitation Assay
TCM: Traditional Chinese Medicine
TJ-48: Shi-quan-da-bu-tang
TUNEL: terminal deoxynucleotidyl transferase-mediated dUTP-biotin nick-end labeling
